# Australian, Irish, and Swedish women’s comfort levels when breastfeeding in public

**DOI:** 10.1186/s12889-023-17472-z

**Published:** 2023-12-18

**Authors:** Louise Gallagher, Vivienne Brady, Lesley Kuliukas, Charlotta Dykes, Christine Rubertsson, Yvonne L. Hauck

**Affiliations:** 1 School of Nursing and Midwifery, Trinity College Dublin, Dublin, D02 T283 Ireland; 2 School of Nursing, Curtin University Perth, Perth, WA Australia; 3 Lund and Malmö University Hospital, Lund, Sweden; 4 Perinatal and Sexual Health, Department of Health Sciences, Medical Faculty, Lund University, Lund, Sweden

**Keywords:** Breastfeeding in public, Comfort, Mothers, Experiences

## Abstract

**Background:**

Despite a flux of global initiatives to increase and sustain breastfeeding rates, challenges persist. The decision to commence and sustain breastfeeding is influenced by multiple, complex factors. Feelings of social embarrassment, shame, fear of judgement, and lack of confidence when breastfeeding in public, compound women’s decisions to breastfeed and may result in formula feeding or early cessation of breastfeeding. A greater understanding of where and how women feel most comfortable when breastfeeding in public can assist in designing interventions to support the initiation and continuation of breastfeeding.

**Methods:**

A cross-sectional survey was conducted with women living in Australia (n = 10,910), Sweden (n = 1,520), and Ireland (n = 1,835), who were currently breastfeeding or who had breastfed within the previous two years. Our aim was to explore where, and how often women breastfeed in public and to compare their levels of comfort when breastfeeding in public. Data were collected in 2018 using an anonymous online survey over a four-week period in Ireland, Australia, and Sweden, and were analyzed using SPSS Version 25.

**Results:**

Most respondents were highly educated, with over 70% in each country reporting having a university or college degree. Observing women breastfeeding in public was more commonly reported to be a weekly or daily occurrence in Sweden (24.5%) and Australia (28%), than in Ireland (13.3%). Women in the participating countries reported breastfeeding in public most commonly whenever their babies needed feeding. Very few women never or rarely breastfed publicly. Coffee shops/cafes, restaurants, and parks were the most popular locations. In all three countries, partners were reported to be very supportive of breastfeeding in public, which enhanced breastfeeding women’s comfort levels. When asked to score out of a maximum comfort level of 10, women reported higher mean levels of comfort when breastfeeding in front of strangers (Ireland M = 7.33, Australia M = 6.58, Sweden M = 6.75) than with those known to them, particularly in front of their father-in-law (Ireland M = 5.44, Australia M = 5.76, Sweden M = 6.66 out of 10), who scored lowest in terms of women’s comfort levels.

**Conclusion:**

This study offers important insights into the experiences and comfort levels of women breastfeeding in public. Limitations include the anonymous nature of the surveys, thus preventing follow-up, and variances in terminology used to describe locations across the three settings. Recommendations are made for research to determine the relationships between the frequency of breastfeeding in public and breastfeeding women’s perceived comfort levels, the influence of family members’ perceptions of breastfeeding in public and women’s experiences, and the experience of women who feel uncomfortable while breastfeeding in public, with a view to developing support measures.

## Background

Despite initiatives to increase breastfeeding rates, many countries continue to have poor initiation rates [1]. In high-income countries such as Sweden, Australia, and Ireland, breastfeeding is more common among educated women than among low-income groups, with fewer years spent in education [2]. Hence, infants most vulnerable to poor nutrition [3] and too few infants, receive the maximum benefits of breastfeeding, and are exposed to risks associated with formula feeding [4]. Women are also missing out on the health benefits associated with breastfeeding, such as lower rates of breast [5] and ovarian cancer [6], along with a reduction in maternal risk of cardiovascular disease [7].

Social embarrassment and challenges around breastfeeding in public have been cited by women as reasons for both choosing to formula feed [8] and for the early cessation of breastfeeding [9]. Evidence suggests that women may choose to formula feed from birth because they fear social disapproval while breastfeeding in public [10]. Pregnant women have described the public side of breastfeeding as “off putting” and expressed concern that breastfeeding may be offensive to members of the public [11]. In a comparison of four European countries (Sweden, Spain, Scotland, and Italy) [12], it was noted that those mothers who had a negative attitude towards breastfeeding in public were less likely to have ever breastfed in a public place and those who had never breastfed in public discontinued earlier than those with positive attitudes.

Fear and social embarrassment around breastfeeding in front of others are often underscored by mainstream and social media reports of women being shamed or told they cannot breastfeed in a public place [13, 14]. Such media discourse can undermine women’s confidence and may contribute to women considering that breastfeeding in public is a problematic practice [9].

Women who choose to breastfeed have highlighted avoiding public breastfeeding in certain places and in front of people with whom they feel uncomfortable [9]. This behaviour may lead to social isolation or prompt a decision to use infant formula or expressed breastmilk instead of breastfeeding when away from the home [12].

While breastfeeding initiation and duration are influenced by many factors [15], Amir [16] suggests that comfort levels while breastfeeding in front of others, may be an important predictor of sustaining breastfeeding. Therefore, to adequately support breastfeeding in different settings, it is important to understand where women actually breastfeed and their comfort levels while breastfeeding in front of others. A greater understanding of the views and experiences of women will assist in designing interventions to improve comfort levels and support the initiation and continuation of breastfeeding.

The aim of this study was to assess where, how often and with what level of comfort, women living in Australia, Ireland and Sweden breastfeed in public.

## Methods

We used an anonymous, self-administered online survey to evaluate where women breastfeed, and with what frequency. We also explored their comfort levels while breastfeeding in front of other individuals. The survey was derived from the literature, and permission was granted by Roche et al. [17] to adapt an existing survey instrument. In addition to collecting demographic data on maternal age, education level, and past and current breastfeeding practices, specific questions were included regarding the frequency of breastfeeding in public, locations, comfort levels generally in front of particular people, partner support, and how often they observed other mothers breastfeed in public. Likert scales asked women to rate their comfort levels from 1 to 10, where 1 indicated very uncomfortable, and 10 indicated very comfortable.

Ethical approval to conduct the study was granted by Curtin University Human Research Ethics Committee in Australia (HRE2018–0037), Research and Ethics Committee, School of Nursing and Midwifery, Trinity College in Ireland (COM_35_17/18), and the Advisory Committee for Research Ethics in Health Education Lund University in Sweden (Reference Number 50– 18). Social media was used to invite women who were living in Australia, Ireland, or Sweden who were currently breastfeeding or had breastfed within the previous two years to participate in the online survey. The online survey was presented on a user-friendly platform suitable for completion on mobile phones (Qualtrics in Australia, SurveyMonkey in Ireland, and Sweden). The recruitment methods for this survey have been described elsewhere [18] as part of a larger cross-sectional study. Data were collected over a four-week period in 2018 in each country (March in Australia, April in Ireland, and December in Sweden).

### Data analysis

Data analysis included one-way ANOVA and descriptive statistics such as frequencies, means, medians, modes and ranges using SPSS version 25.

## Results

The characteristics of the 10,910 women living in Australia, 1,520 living in Sweden and 1,835 living in Ireland are presented in Table 1. The mean age of women living in Ireland was slightly older (34.9 years) than women participating in Australia (32.3 years) or Sweden (32.8 years). Respondents were predominantly highly educated, with 89.2% of Irish women, 82.4% of those from Sweden and 70.6% from Australia reporting that they had a university or college degree. The largest proportion of women responding in each country gave birth to one child (42% in Ireland, 46% in Australia and 48% in Sweden).

**Table 1:** Demographic characteristics of women living in Ireland, Australia and Sweden

Demographic characteristics	Ireland	Australia	Sweden
Maternal age			
(N = 10,885 Australia, N = 1831 Ireland, N = 1520 Sweden)	34.9 mean 35 median 35 mode Range 18 to 49 (SD 4.109)	32.3 mean 32 median 33 mode Range 17 to 53 (SD 4.79)	32.8 mean 32 median 32 mode Range 18 to 57 (SD 4.92)
Highest education level			
(N = 10,910 Australia, N = 1820 Ireland, N = 1520 Sweden)			
University / college postgraduate degree University / college undergraduate degree High / secondary school completed High / secondary school not completed	943 (51.8%) 681 (37.4%) 183 (10.1%) 13 (0.7%)	2997 (27.5%) 4707 (43.1%) 2623 (24.0%) 583 (5.3%)	835 (55.0%) 417 (27.4%) 252 (16.6%) 16 (1%)
Number of children			
(N = 10,910 Australia, N = 1800 Ireland, N = 1409 Sweden)			
1 child 2 children 3 children 4 or more children	761 (41.9%) 664 (36.6%) 226 (15.0%) 109 (6.4%)	4990 (45.7%) 4097 (37.6%) 1341 (12.3%) 482 (4.4%)	677 (48.0%) 537 (38.0%) 150 (11%) 45 (3%)

### Patterns of breastfeeding in public during current or last breastfeeding experience

A total of 14,247 women responded to questions regarding the frequency of breastfeeding in public and the actual place where they breastfed, during their most recent breastfeeding experience. Table 2 demonstrates that most women in the participating countries breastfed in public whenever their babies required feeding, and very few breastfeeding women never or rarely breastfed in public. Observing other women breastfeeding in public was not a daily occurrence in any of the three countries. Irish mothers were less likely to report seeing other mothers breastfeed in public, compared to women living in Australia or Sweden. There were some differences in the locations where women breastfed among the three countries, but coffee shops/cafes, restaurants, and parks were the most prevalent responses.

**Table 2:** Patterns of breastfeeding in public

During current or last breastfeeding experience, how often did you breastfeed in public	Ireland (N = 1821)	Australia (N = 10,906)	Sweden (N = 1520)
whenever my baby needed to be fed	1313 (71.6%)	7521 (69.0%)	69 (65.7%)
between 1 & 5 times a week	203 (11.4%)	1207 (11.1%)	14 (13.3%)
between 1 & 5 times a month	211 (11.7%)	1316 (12.1%)	15 (14.3%)
rarely	89 (4.8%)	734 (6.7%)	6 (5.7%)
never	5 (0.4%)	128 (1.2%)	1 (1%)
Thinking about the past year, how often have you seen other mothers breastfeed in public			
daily	32 (1.8%)	429 (4.3%)	5 (4.9%)
between 1 & 5 times a week	209 (11.5%)	2371 (23.7%)	20 (19.6%)
between 1 & 5 times a month	629 (34.4%)	3948 (39.4%)	44 (43.1%)
rarely	896 (49.3%)	3194 (31.9%)	33 (32.3%)
never	53 (3.1%)	70 (0.7%)	0 (0%)
Places you do or did breastfeed in public			
Restaurant /coffee shop/cafe	1786 (97.2%)	9839 (90.2%)	87 (87%)
Park	1259 (68.5%)	9702 (88.9%)	72 (72%)
Shopping centre / mall	1601 (87.1%)	9439 (86.5%)	84 (84%)
Parent group	0	7513 (68.9%)	69 (69%)
Health centre	1276 (69.4%)	7218 (66.2%)	76 (76%)
Baby clinic	35 (1.9%)	5984 (54.8%)	0
Library	396 (21.6%)	4440 (40.7%)	47 (47%)
Breastfeeding support group	1192 (64.9%)	2624 (24.1%)	9 (9%)
Place of worship	553 (30.1%)	1954 (17.9%)	29 (29%)

Irish women were more likely to report breastfeeding at a dedicated breastfeeding support group (65%) than their Australian or Swedish (9%) counterparts. Some of the terminology used in this survey, such as ‘parent group’ in Ireland, and ‘baby clinic’ in Sweden, may not have been familiar to women in a particular country and may account for the nil responses.

### Comfort levels while breastfeeding in public

In addition to questions regarding patterns of breastfeeding in public, women were asked to rate support from their partners regarding breastfeeding in public and their overall comfort level while breastfeeding in public. In all three countries, partners were found to be very supportive of breastfeeding in public (Table 3). Overall comfort levels while breastfeeding in public were lower (average 7) than those for comfort in front of their partner but were also consistent among the three countries. Women in Ireland reported slightly higher levels of overall comfort (7.47) with breastfeeding in public, than those in Sweden and Australia (7.17).

**Table 3:** Partner support for breastfeeding in public and overall reported comfort level

	Ireland		Australia		Sweden	
	M	SD	M	SD	M	SD
How supportive was your partner with your decision to breastfeed in public	9.57 (N = 1759)	1.24	9.22 (N = 9817)	1.86	9.57 (N = 1520)	1
During your last breastfeeding experience overall how comfortable were you to breastfeed in public	7.47	2.33	7.17	2.53	7.17	2.66

Rated from 1–10 (10 = very comfortable)

Women were also asked to rate their personal comfort levels while feeding in front of their partners and other individuals from their family, friends, and peers. The highest levels of comfort were reported when feeding in front of partners in all three countries. In relation to other individuals, women scored their father-in-law lowest in terms of comfort levels while breastfeeding. Swedish women reported higher levels of comfort when feeding in front of their father-in-law (6.66) than Irish (5.44) and Australian women (5.76). Comfort levels were very consistent among the three countries. Irish women reported higher comfort levels in front of strangers (7.33) than their fathers (7.31), fathers-in-law (5.44), other relatives (6.87), and male friends (6.2) (Table 4). Australian and Swedish women also reported higher comfort levels while feeding in front of strangers than their fathers-in-law.

**Table 4:** Comfort levels while feeding in front of others

During your last breastfeeding experience how comfortable were you to breastfeed in front of:	Ireland Mean	Australia Mean	Sweden Mean
Partner	9.67	9.69	9.93
Other BF mothers	9.63	9.29	*
Mother	8.96	9.1	8.67
Female Friends	8.77	8.81	9.1
Health Professionals	8.89	8.53	8.58
Other parents	7.88	7.95	8.33
Mother-in-law	7.37	7.71	7.73
Father	7.31	7.45	7.76
Other relatives	6.87	7.08	7.18
Strangers	7.33	6.58	6.75
Male Friends	6.2	6.25	6.96
Father-in-law	5.44	5.76	6.66

*Missing data

1 = very uncomfortable, 10 = Very comfortable

A one-way ANOVA was performed to determine if there were differences between the means of any categories in the three countries. No statistically significant differences were observed (F [3, 29] = 1.522, p = 0.96) among the three countries for any of the categories.

## Discussion

Our survey includes over fourteen thousand highly educated women in three countries with both high (Sweden 94%, Australia 95.9% [31, 32] and low initiation rates (Ireland 62%, [33]). It is plausible to consider that such differences in rates would be grounded in cultural and traditional views around breastfeeding, which may reflect initiation rates. Ireland, Sweden, and Australia all have legislation in place providing maternity leave and protecting the mothers’ right to breastfeed publicly [34]; however, each has distinct differences in maternity leave entitlements (18 weeks paid in Australia [35], Ireland 26 weeks paid, Sweden 480 days (68 weeks)) [34], which may impact breastfeeding rates and practices. Sweden, while offering generous maternity leave, also provides some incentives for fathers to take some of the leave that is allotted to both parents. This has been suggested as a possible reason for the declining breastfeeding continuation rates in Sweden in recent years [35]. Ireland, while also providing paid maternity leave for up to six months, has high rates of women engaged in part-time work but a lack of state support for childcare, meaning that many women may face challenges of working while simultaneously attending to childcare roles, which may impact on rates of initiation and duration [36]. Australia has the shortest maternity leave provision among the three countries, a factor that has been found to contribute to shorter durations of breastfeeding and increased introduction of formula [37]. Proposed changes to Australian legislation would increase paid parental leave by two weeks each year until 26 weeks in 2026 [35]. This survey highlights that although cultural contexts may vary, women share similar rates of comfort while breastfeeding publicly.

Two thirds of this sample of Irish, Australian, and Swedish breastfeeding women report breastfeeding in public whenever their baby needed to be fed, suggesting that this is a relatively common practice. However, it was also evident that some women never or rarely breastfed in public (Ireland: 5%; Sweden: 7%; Australia: 7%), suggesting that they either used alternative feeding methods or were socially isolated. Restricted freedom and social isolation are important concepts that have been identified as barriers to breastfeeding [19] and contribute to early cessation [20]. The introduction of alternative feeding methods has also been determined to be a risk factor for the cessation of breastfeeding [21]. It was not possible due to the anonymous nature of our survey, to determine the relationship between the frequency of breastfeeding in public and perceived comfort levels while breastfeeding in front of others, but these are concepts that require further study to determine whether they are interrelated, or impact on women’s experiences. In a thematic synthesis of 71 studies, Grant et al. [22] identified that women exhibited highly self-aware behaviours while breastfeeding in public, which led to ‘discrete’ breastfeeding or avoiding it altogether, which served to protect them from the opinions and attitudes of others. This synthesis suggested reframing breastfeeding in public to place less emphasis on the woman and consider the needs of the baby as paramount.

Despite evidence published from our wider survey that breastfeeding in public is often challenging for breastfeeding mothers [23], women in all three countries reported generally high levels of comfort while breastfeeding in public. This common experience is underscored by high levels of support from their partners relating to this practice. Discomfort while breastfeeding in public has previously been associated with shortened lactation duration in other studies [21, 22]. Our study results suggest that women who breastfeed in public are comfortable doing so and hence strategies need to be devised support those who plan to breastfeed in public so that social isolation is not associated with breastfeeding and barriers and challenges can be mitigated against.

The reported levels of maternal comfort may be predicated by women in this study, having predominately high levels of education. Australia and Sweden were ranked 1st and 3rd respectively, among OECD countries for the number of years spent in education, and Ireland is ranked 10th among the 41 OECD member countries [38]. Education has consistently been noted as a predictor of breastfeeding initiation in industrialised nations [39]. High levels of education in this sample, including postgraduate studies, may also have contributed to comfort levels while breastfeeding in public. Such high comfort levels may not be shared by women with fewer educational opportunities and lower socioeconomic backgrounds, who have been consistently found to have lower levels of initiation and duration than other mothers.

Despite predominantly high levels of comfort while breastfeeding, a small proportion of women in each country did not score high comfort levels while breastfeeding in front of their partners, indicating that they do not feel universally accepted. In a qualitative review of barriers and enablers to public breastfeeding, Grant et al. [22] identified that partners often expressed concern over public breastfeeding as they feared confrontation with strangers. Bennet et al. [25] also noted that one in ten Irish fathers felt uncomfortable with an unrelated woman breastfeeding in public, which increased to one third of fathers if their partner breastfed in public. The fathers of breastfeeding partners (n = 417) who participated in this survey were predominantly married (87%), working full-time (92%), and had a college degree (77%). Specific concerns regarding their partner breastfeeding in public were identified, including disapproving looks and leering from other people, particularly other men [25]. Importantly, our data suggest that for the most part any partner concern or discomfort is not generally felt by the breastfeeding woman, but still exists. This evidence could be used to devise interventions and educate partners about their role in supporting breastfeeding. A systematic review of perinatal interventions aimed at enhancing breastfeeding among male partners by Abbass-Dick et al. [26], identified that culturally appropriate interventions may be beneficial for increasing breastfeeding outcomes. Future designs of interventions should consider providing face-to-face delivery and including partners [26] to provide education on how to support breastfeeding in public, so that women feel supported, and partners are given strategies and practical tips on how to offer that support.

Women reported feeling most uncomfortable breastfeeding in front of their fathers-in-law contrasting with data from other studies in the USA, Australia and Canada, which have found that females are more likely to consider public breastfeeding as offensive, rather than men [27, 28]. Negative thoughts about breastfeeding in public have previously been attributed to views of other family members [22], particularly among marginalised breastfeeding women [29]. Although grandmothers have been identified as important to the women’s breastfeeding experience [30], to-date, little has been published on the influence of grandfathers. In our study, we focused on the woman’s own comfort level; therefore, it is unknown whether this discomfort was reciprocal on the part of the father-in-law. It could be that familial relationships and ‘kinship’ may be experienced to a lesser degree with fathers-in-law than their own fathers, or their mothers-in-law with whom they may share more intimate experiences. Although grandfathers often make contributions to the care of their grandchildren alongside grandmothers, their voices and data pertaining to them are frequently missing from any research into their role [38]. Our data suggest that further research on the perceptions of grandfathers regarding breastfeeding is urgently required, as this may have an important bearing on women’s experiences.

In our survey, we found considerable congruity between women’s levels of comfort while breastfeeding in public across all three countries. In keeping with the lower rates, women in Ireland were however less likely to see other women breastfeeding publicly. Vicarious exposure to breastfeeding in public has been identified as a determinant of a positive breastfeeding attitude and may enhance women’s breastfeeding self-efficacy [39]. Low initiation rates in Ireland may impact the overall cultural acceptance of breastfeeding, which has been lacking a breastfeeding culture for several generations [25]. Women have reported that seeing other women breastfeed in public as a normal part of life is critical to establishing a supportive breastfeeding culture [34]. The results from our survey show that women from three diverse contexts, with varying rates of breastfeeding initiation, generally feel comfortable while breastfeeding and this information could be useful to those women who report choosing formula feeding due to perceived negative perceptions of others [17]. Alternatively, it may be possible that women who do choose to breastfeed publicly already have high breastfeeding self-efficacy and therefore further research is required to explore the experiences of women who feel uncomfortable while breastfeeding in public in order to be able to tailor support to enhance their experience.

Finding a space for breastfeeding outside of the home, has been identified as challenging in other work [41]. Our study shows that women breastfeed most frequently in restaurants or cafés. Evidence suggests that some women are not in a financial position to use spaces such as coffee shops and restaurants [42], therefore these locations may not be accessible to all. Boyer [41] noted that public breastfeeding spaces often vary between high-and low-income areas, creating constraints for some women in terms of accessible places to breastfeed.

Overall, our findings highlight that, for the most part, Irish, Swedish, and Australian women feel comfortable while breastfeeding in public. They also perceive positive support from their partners and breastfeed in a variety of public spaces; however, breastfeeding in the presence of others, particularly fathers-in-law, decreases comfort levels. Seeing other women breastfeed in public did vary between the countries and was more common in Sweden and Australia than in Ireland, which may be important for women’s vicarious experiences of breastfeeding.

### Limitations

The limitations to our survey include some of the terminology used to describe the locations where women breastfeed. Due to the nil responses for parenting groups and baby clinics, it is apparent that either these groups or locations were not accessible to them, or they did not exist in that context. It is also plausible that some of the terminology used was unfamiliar to women. Additionally, we did not provide a definition of breastfeeding in our survey; therefore, it is possible that women who provided expressed breastmilk via a bottle may have contributed to our findings.

Our survey is also skewed demographically. The sample consists of highly educated women, living in high-income countries and may not be representative of women from diverse backgrounds and different contexts. Further research is warranted to understand the comfort levels of younger women from lower socioeconomic backgrounds to garner their views regarding breastfeeding in public.

## Conclusion

This research offers important insights into the experiences and comfort levels of highly educated women breastfeeding in public in 3 high income countries. Women indicated overall high levels of comfort while breastfeeding in public; however, there are important findings in relation to some family members that require further research. There is a need for further insights into the perceptions of the women’s wider family regarding breastfeeding, so that interventions can be specifically targeted at ensuring that women feel comfortable while breastfeeding in public.

## Data Availability

Three separate SPSS databases were used in this study (Australian, Irish and Swedish). These data were not merged as the Swedish transcripts and SPSS data file are not available in English and only descriptive statistics were performed. The datasets used and/or analysed during the current study are therefore not reposited but are available from the corresponding author on reasonable request.
